# Aged black tea alleviates constipation in mice by modulating intestinal neurotransmitters and decreasing AQP3 and AQP9 expression

**DOI:** 10.29219/fnr.v67.9513

**Published:** 2023-10-30

**Authors:** Yu Wu, Qiuhua Li, Junxi Cao, Fenling Fan, Lishe Gan, Rihui Wu, Jingwei Jin, Ruohong Chen, Lingli Sun, Zhenbiao Zhang, Xingfei Lai, Wing-Leung Wong, Shili Sun, Dongli Li

**Affiliations:** 1 School of Biotechnology and Health Sciences, Wuyi University, Jiangmen, China; 2 Tea Research Institute, Guangdong Academy of Agricultural Sciences / Guangdong Provincial Key Laboratory of Tea Plant Resources Innovation & Utilization, Guangzhou, China; 3 International Healthcare Innovation Institute (Jiangmen), Jiangmen, China

**Keywords:** aged black tea, constipation, intestinal neurotransmitter, AQP3, AQP9

## Abstract

**Background:**

Black tea is fully fermented tea with abundant functional components that benefit the gastrointestinal tract. But whether black tea extract relieves constipation is unknown. Therefore, we used loperamide to induce constipation in mice to assess the therapeutical effect of extracts from aged black tea with different storage times.

**Design:**

Sixty-three C57BL/6J male mice were randomly divided into Control group (Con), Model group (Mod), Positive group (Pos), aged 6 years group (15Y), aged 4 years group (17Y), aged 2 years group (19Y), and unaged group (21Y). Mice were given loperamide (20 mg/kg, twice a day) to induce constipation for 10 days, and black tea extracts (500 mg/kg) were intragastrically given for 7 days while continuing modeling.

**Results:**

The results showed that black tea extracts relieved constipation symptoms by improving defecation weight, fecal water content, and gastrointestinal transit rate. Black tea extracts can also protect colon tissue, regulate serum neurotransmitters, increase the levels of excitatory neurotransmitters motilin (MTL) and substance P (SP), and decrease the levels of inhibitory neurotransmitters vasoactive intestinal peptide (VIP) and nitric oxide (NO). Immunohistochemistry (IHC) showed that black tea extracts were able to reduce AQP3 and AQP9 expression in the colon of constipated mice. In addition, Reverse transcription-quantitative polymerase chain reaction (RT-qPCR) showed that black tea extracts could decrease AQP3 and AQP9 mRNA expression. The relief effect of aged black tea (15Y) with the longest storage was better than that of other years, which may be due to the role of active ingredients such as thearubigins (TRs), soluble sugar, tea polysaccharide (TPS), gallic acid (GA), and catechin gallate (CG) in aged black tea.

**Conclusions:**

Based on these results, we believe that regular consumption of black tea is effective in relieving constipation, and that black tea is more effective in relieving constipation as the storage time increases.

## Popular scientific summary

- The results showed that black tea extracts relieved constipation symptoms, and can also regulate serum neurotransmitters, reduce AQP3 and AQP9 expression in the colon of constipated mice.
- Black tea is more effective in relieving constipation as the storage time increases, which may be due to the role of active ingredients such as TRs, solule sugar, TPS, GA and CG.
- The results indicate that aged black tea could be used as an effective dietary strategy to relieve constipation.

The symptoms of abdominal discomfort are often associated with constipation, a common digestive disorder. The main clinical manifestations of constipation are persistent difficulty in defecation, reduced defecation, or feeling of incomplete defecation (1). With the increasing mental stress of social people and the continuous emergence of Western-style diet, the surge of the aging population, the probability of constipation symptoms will increase over the age of 65, its incidence is increasing, and it develops towards younger age. Approximately 16% of all adults of all ages have this disease, and it severely affects their quality of life (2, 3). The key to reducing chronic constipation’s negative effects is to find a way to alleviate it effectively.

Despite the fact that constipation’s etiology is multifactorial, its pathophysiology is not yet fully understood. The occurrence of constipation is currently considered to be mainly related to colorectal motility disorders, psychosocial factors as well as abnormal gastrointestinal regulatory neurotransmitters, and abnormal colonic aquaporin expression (4, 5). There are dozens of known intestinal neurotransmitters, among which excitatory neurotransmitters such as substance P (SP), acetylcholine (ACh), motilin (MTL) and inhibitory neurotransmitters such as vasoactive intestinal peptide (VIP), and nitric oxide (NO) are the most intensively studied neurotransmitters. MTL is considered to be a brain-gut peptide that initiates gastrointestinal contractile activity and is closely related to gastrointestinal motility disorders (6). SP is an endogenous polypeptide involved in cell proliferation, migration, and immune regulation and can sustain intestinal structure more intact (7). VIP is an important regulator of intestinal peristalsis that can relax gastrointestinal smooth muscle (8). NO was found to mediate nonadrenergic, noncholinergic inhibitory nerves and play an important role in colonic dysmotility of patients with slow-transit constipation (9).

Aquaporins (AQPs) play a pivotal role in the regulation of intestinal absorption, secretion, and water metabolism by mediating the transmembrane transport of water molecules (10). Among them, AQP3 and AQP9 are important proteins that are associated with constipation. AQP3 is expressed in the colon and predominantly in mucosal epithelial cells; it is considered to be one of the most important functional molecules in water transport within the colon (11). Upregulation of AQP3 expression in colonic epithelial cells can lead to severe constipation (5). AQP9 is specifically found in goblet cells and is involved in the synthesis and/or secretion of a certain mucus, which may protect the intestinal surface and smooth the flow of intestinal contents (12). Some studies have suggested that rats with constipation may have a greater absorption of water in their colons and less secretion of intestinal fluid because of active expression of AQP3 and AQP9 concluded that a significant increase in AQP3 and AQP9 in rats’ colon resulted in intestinal smooth muscle spasms, and a significant increase in water absorption, affecting the frequency of intestinal peristalsis, and leading to constipation (13, 14). Hu et al. found increased AQP3 and AQP9 contents in the colon of mice with constipation induced by diphenoxylate (15).

Laxatives, especially osmotic or secretory laxatives, are being used often to treat constipation; however, patients often have negative effects from using these medications (16, 17). Functional food generally offers better health benefits and safety compared to medicines (18).

China is the largest producer and consumer of tea in the world and has abundant tea plant resources and tea products. Tea contains a variety of active ingredients with unique medicinal and healthcare functions and has broad development prospects as a health food and drug. The phytochemical components of tea are involved in the prevention and cure of many illnesses like cardiovascular diseases, malignancy, digestive dysfunction, and metabolic disorders like obesity and diabetes (19). Black tea is one of the six kinds of tea in China because of its unique aroma and taste quality. At present, black tea accounts for more than 70% of the world’s tea production, trade, and consumption (20). The polyphenols and pigments in black tea are highly beneficial to the human body and have a good health care effect. It has been shown in the literatures that yellow tea and Pu-erh tea have anticonstipation effects in mice (8, 21). But there is no relevant study report of the therapeutical effect of extracts from black tea on loperamide-induced constipation in mice.

Aged tea refers to processed tea stored for more than 2 years. Aged tea has been admired by tea guests because of its natural health care efficacy. Aged tea is called ‘healthy beverage’ because modern scientific research shows that it is rich in nutrients and pharmacodynamic components. Catechins, flavanols, and their oxidative products in aged tea are a complex class of physiologically active substances and play an important role in human health (22). Storing aged tea has very important significance: 1) with the increase of storage years, the content of active ingredients in tea has changed, which may result in better health effects; 2) with the increase of storage years, it leads to better quality of some teas, especially aged green tea, with reduced bitterness and astringency; 3) aged tea can increase the production value, after a few years of storage, aged tea gradually becomes more and more highly sought, and its price may be even higher than new tea. Therefore, in the present study, Ying Hong No. 9 black tea with different aging periods (producted in 2015, 2017, 2019 or 2021) was selected to assess the effects on intestinal propulsion rate, colonic lesions, serum neurotransmitter levels, and colonic hydroprotein AQP3 and AQP9 expression levels in mice by establishing a loperamide hydrate-induced constipation model in order to assess the anticonstipation effect of black tea and investigate whether the storage of black tea affects its health benefits, which may provide a theoretical basis for the scientific consumption of aged black tea driven by health attributes.

## Materials and methods

### Preparing black tea extracts

We obtained samples of black tea from the cultivar Yinghong NO.9 (Tea Research Institute, Guangdong Academy of Agricultural Sciences, China). The fresh leaves of Ying Hong No. 9 tea with one bud and two leaves are used as raw materials and processed into finished tea through withering, kneading, fermentation, and drying processes. The finished tea is stored in temperature 25 ± 5°C, humidity 75 ± 5%, low oxygen, and protected from light.

According to a solid-to-liquid ratio of 1:20, 100 g of tea was added to water at 100°C and maintained there for 60 min before being filtered through two layers of gauze. The filter residue was extracted for 60 min with the same techniques. Aqueous black tea extract was prepared from the combined filtrates, filtered again, freeze-dried, subsequently sealed, and packaged for later use.

### Tea component analysis

Chinese National Standards (GB/T 8303-2013; GB/T 8305-2013) were used to determine the moisture and water extract content in tea leaves. Spectrophotometry was used to determine the content of tea polyphenols in tea leaves (GB/T 8313-2018). Total free amino acids in tea were determined by the ninhydrin method (GB/T 8314-2013). Total soluble sugars and tea polysaccharides (TPS) were determined by the anthrone-sulfuric acid colorimetric method. Contents of theaflavins (TFs), thearubigins (TRs), and theabrownins (TBs) in tea samples were determined by colorimetry.

### High-performance liquid chromatography

The amount of catechins in tea leaves was measured using high-performance liquid chromatography (HPLC). Dissolve gallic acid (GA), gallocatechin (GC), epigallocatechin (EGC), catechin (C), caffeine (CAFF), epicatechin (EC), epigallocatechin gallate (EGCG), gallocatechingallate (GCG), epigallocatechin gallate (ECG), and catechin gallate (CG) standards in 70% methanol to prepare the corresponding standard solution (100 μg/mL). Black tea leaves were extracted with 70% methanol and filtered through 0.45-μm filter, respectively. Then, tea extracts were analyzed using the Agilent 1260 HPLC system fitted with a Zorbax column (250 mm × 4.6 mm, 5 μm) to determine primary catechins. Flow rate of 1 mL/min was used for the gradient elution of the samples. For gradient elution, acetonitrile (phase A) and 0.05% aqueous phosphoric acid (phase B) were utilized. In the first 30 min of elution, phase A increased from 6.5 to 16%, while phase B decreased from 93.5 to 84%. At 30–34 min of elution time, phase A increased from 16 to 20%, and phase B decreased from 84 to 80% and then continued to 39 min. At 40 min of elution time, phase A decreased to 6.5%, and phase B increased to 93.5% and then continued to 45 min for reequilibration. Samples were injected at a volume of 10 μL and were detected at a wavelength of 280 nm. The temperature was maintained at 30°C during all operations. All the major compounds (Table 1) found in the tea extracts were verified with their corresponding standards using HPLC. In addition, the quantitative analysis was performed by the external standard method according to the peak area.

**Table 1:** Contents (mg/g) of catechin and caffeine monomers in black tea samplesa

Component	15Y	17Y	19Y	21Y
C	0.896 ± 0.134^c^	1.082 ± 0.012^bc^	2.3 ± 0.061^a^	1.25 ± 0.062^b^
CG	1.979 ± 0.336^a^	0.561 ± 0.035^b^	1.617 ± 0.017^a^	0.52 ± 0.002^b^
EC	1.475 ± 0.065^c^	1.791 ± 0.032^c^	5.827 ± 0.165^a^	2.183 ± 0.159^b^
ECG	6.69 ± 1.113^b^	5.655 ± 0.008^b^	15.433 ± 0.047^a^	5.66 ± 0.061^b^
EGC	3.26 ± 0.12^c^	5.8 ± 0.29^b^	8.11 ± 0.4^a^	7.95 ± 0.17^a^
EGCG	5.01 ± 0.143^c^	7.679 ± 0.003^a^	5.662 ± 0.37^b^	7.286 ± 0.058^a^
GC	56.964 ± 1.894^a^	15.516 ± 0.235^c^	52.181 ± 0.245^b^	16.629 ± 0.112^c^
GCG	2.007 ± 0.18^b^	1.725 ± 0.003^b^	2.383 ± 0.021^a^	1.85 ± 0.089^b^
GA	1.93 ± 0.094^a^	1.756 ± 0.047^a^	1.483 ± 0.049^b^	1.248 ± 0.072^c^
CAFF	55.92 ± 1.4^b^	54.47 ± 0.03^b^	55.92 ± 0.3^b^	59.047 ± 0.729^a^

a Dissolve gallic acid (GA), gallocatechin (GC), epigallocatechin (EGC), catechin (C), caffeine (CAFF), epicatechin (EC), epigallocatechin gallate (EGCG), gallocatechingallate (GCG), epigallocatechin gallate (ECG), catechin gallate (CG); Values represent mean ± SD (*n* = 3). Different letters (a, b, c, d) in the same row indicate significant differences between mean values (*P* < 0.05).

### Animal experiments

The animal study protocol was conducted in accordance with the Animal Care and Use guidelines of Tea Research Institute Guangdong Academy of Agricultural Sciences and approved by the Institutional Animal Care and Use Committee (protocol no. 2021100). Male C57BL/6J mice (aged 8 weeks) were divided into control group (Con), model group (Mod), positive group (Pos), aged 6 years group (15Y), aged 4 years group (17Y), aged 2 years group (19Y), and unaged group (21Y), with nine mice in each group. Mice were housed at 60–65% humidity and room temperature (23 ± 2°C) under a 12 h light/dark cycle, with unrestricted access to food and drink.

All mice except the normal control group were administered loperamide hydrochloride (20 mg/kg) twice daily for 10 days by gavage after they had been domesticated for a week. Over the next 7 days, the mice were gavaged with loperamide and given intragastrically mosapride (2.5 mg/kg, used as a positive drug) or tea extracts (500 mg/kg) daily, while normal and model mice received the same volume of water. Food and drinking water were changed every 2 days, and the total amount of water intake in each group was recorded.

Water intake per mouse (g) = total amount of water intake in each group / number of mice.

### Test for defecation

On days 9 and 16, defecation was evaluated. The mice were transferred to a spotless feeding cage that had filter paper on top of its bottom. After collecting the feces for a full 24 h, the number of fecal particles was counted. Within 30 min, collect new feces and place them to an aluminum box that has been cleaned and weighed. After weighing the metal box and fecal material, the aforementioned process was repeated three times for each group. Feces were placed in a dryer and dried at 103°C for 2 h before being cooled to room temperature. The following formula is how we calculated the fresh fecal water content and fecal pellet weight:

Water content of fresh feces (%) =

[weight of aluminum box before drying (g) – weight of aluminum box after drying (g)] / [weight of the aluminum box before drying (g) – aluminum box weight (g)] × 100%.

Fresh fecal pellet weight (mg) equals fecal mass (mg) divided by the quantity of feces.

### Gastrointestinal transit rate

Mice were fasted from 9:00 p.m. on the 17th day, with free access to drinking water. After 12 h, the mice were given 0.2 mL of Indian ink as an indication. All mice were anesthetized with urethane 20 min later, and blood was drawn from the retro-orbital venous plexus, mice were killed by cervical dislocation. After dissecting mice, intestinal parts from the stomach to the anus were recovered. Both the ileocecal junction and the pylorus of the gut were measured. Indian ink’s migratory path from the pylorus to the ink edge was measured.

The following formula was used to get the gastrointestinal transfer rates (GI):

Gastrointestinal transit rate (%) = migration distance of Indian ink / length of the intestine × 100%.

### Histology staining

Colon tissues were preserved in 10% formalin solution for 24 h. The paraffin-embedded 3 mm blocks of the fixed colon tissues were cut from the fixed colon tissues. Using a paraffin microtome (Leica, Switzerland), the tissue blocks were divided into 4 μm thick slices that were subsequently deparaffinized twice with xylene and rehydrated with 100, 95, 80, and 70% ethanol in sequence. Hematoxylin and eosin (H&E) staining was applied to the successive sections (Shanghai Beyotime Biotechnology). The stained sections were sealed with neutral resin, dehydrated with 95 and 100% ethanol, twice cleaned with xylene, and then examined under a light microscope (Olympus, Japan). Finally, the colon’s histology was assessed.

### Characterization of neurotransmitters in serum

Twenty minutes were spent centrifuging the obtained blood at 3000 g. Using ELISA kits, the serum concentrations of MTL, SP, VIP, and NO were determined (MEIMIAN).

### Immunohistochemistry (IHC)

After being heated with ethylene diamine tetraacetic acid (EDTA) (Solarbio) to unmask the antigens, paraffin slices were treated with 3% H_2_O_2_ to squelch endogenous peroxidases. The sections were incubated overnight at 4°C with primary antibodies directed against AQP3 (Bioss, BA1559, 1: 200) and AQP9 (Bioss, BA3622-1, 1: 300) after blocking with 5% goat serum (Solarbio) for 30 min. The sections were then probed with the secondary antibody for 1 h, followed by the streptavidin-biotin complex (SABC) reagent (Shanghai Beyotime Biotechnology) for 30 min. After color development with diaminobenzidine (DAB) (Shanghai Beyotime Biotechnology) for 1–2 min, the sections were counterstained with hematoxylin (Shanghai Beyotime Biotechnology) for 90 s, dehydrated through an ethanol gradient, cleared with xylene, mounted with neutral resin, and observed under a light microscope (Olympus, Japan). The images were analyzed using the Image J software.

### Quantitative PCR detection of aquaporin mRNA expression

Mice underwent colectomies and colons were stored at −80°C. Total RNA was extracted using the RNA extraction kit (OMEGA) as indicated by grinding with grinding beads. With the use of a UV (ultraviolet) spectrophotometer, the RNA’s purity was evaluated. The extracted total RNA was then synthesized into cDNA by a reverse transcription reaction kit (TOYOBO). Reverse transcription products were measured in a PCR instrument (ROCHE) using primers listed in Table 2 configured according to the loading method described in the instructions for Hieff^®^ qPCR SYBR Green Master Mix (No Rox) (YASEN). Threshold cycle number (Ct) and relative copy quantification of the samples were obtained by the analysis software and then compared between samples. Data analysis was performed using the 2^-ΔΔCt^ method.

**Table 2:** Nucleotide sequences of primers

Gene name	Sequence (5′–3′)	Length/bp
AQP3	Forward: GCTGTGACCTTCGCAATGTG Reverse: CAAAGATTCCAGCTGTGCCG	20
AQP9	Forward: CGAGAAAAGGCTGGTGGGAT Reverse: AAAAGACACCGCTGGGTTGA	20
GAPDH	Forward: GGAGAGTGTTTCCTCGTCCC Reverse: ACTGTGCCGTTGAATTTGCC	20

### Statistical analysis

The mean and standard deviations are used to represent the data (mean ± standard deviation [SD]). Software called GraphPad Prism 8.0 was utilized for the statistical evaluation. One-way Analysis of Variance (ANOVA) with Duncan’s new multiple-range test (MRT) was used to compare the median values for each group to see whether there were any differences. Statistics were deemed significant at *P* < 0.05.

## Results

### Composition of the tea extracts

We first analyzed black teas from different years, including four batches stored since 2015 (15Y), 2017 (17Y), 2019 (19Y) and 2021 (21Y). As demonstrated in Table 3, 15Y has a considerably larger concentration of water, water extract, soluble sugar, and TRs than the other samples when compared the traditional composition of the teas. The levels of amino acids and flavonoids in sample 21Y were substantially greater than those in other samples. TPS had a greater concentration after a longer aging period (15Y: 6-year-aged) than 17Y or 21Y. 19Y had a substantially greater concentration of polyphenols than other samples. Long-aged black teas (15Y: 6-year-aged, 17Y: 4-year-aged) contained more TBs than 19Y or 21Y. Furthermore, there was no significant difference between the groups in terms of TFs.

**Table 3:** Contents (%) of main phytochemical components in black tea samplesa

Component	15Y	17Y	19Y	21Y
Water	11.05 ± 0.05^a^	10.33 ± 0.05^b^	8.62 ± 0.06^d^	9.41 ± 0.02^c^
Water extract	37.78 ± 0.1^a^	36.3 ± 0.01^b^	36.48 ± 0.07^b^	34.97 ± 0.18^c^
Flavonoids	1.47 ± 0.01^c^	1.56 ± 0.03^b^	1.40 ± 0.02^d^	1.72 ± 0.00^a^
Amino acids	2.26 ± 0.02^c^	1.9 ± 0.02^d^	2.6 ± 0.07^b^	2.71 ± 0.05^a^
Soluble sugar	7.47 ± 0.09^a^	6.97 ± 0.06^b^	6.5 ± 0.00^c^	6.26 ± 0.02^d^
Tea polysaccharides	1.01 ± 0.09^a^	0.86 ± 0.04^b^	0.93 ± 0.04^ab^	0.86 ± 0.02^b^
Tea polyphenols	12.77 ± 0.03^b^	12.39 ± 0.07^c^	16.87 ± 0.00^a^	11.36 ± 0.1^d^
Theaflavins	0.13 ± 0.00^a^	0.10 ± 0.00^b^	0.11 ± 0.01^ab^	0.11 ± 0.01^ab^
Thearubigins	2.19 ± 0.01^a^	1.63 ± 0.17^c^	1.70 ± 0.02^b^	1.42 ± 0.01^d^
Theabrownins	3.35 ± 0.06^a^	3.46 ± 0.03^a^	2.69 ± 0.03^c^	2.93 ± 0.03^b^

a Values represent mean ± SD (*n* = 3). Different letters (a, b, c, d) in the same row indicate significant differences between mean values (*P* < 0.05).

The active ingredients in tea such as polyphenols and catechins are believed to be helpful in the treatment of gastrointestinal disorders. The components of the main active ingredients obtained from the tea extracts were analyzed by HPLC. As indicated in Table 1, for the examination of catechins, gallic acid, and caffeine, 19Y had considerably greater levels of C, EC, ECG, and GCG than other samples. 15Y and 19Y had considerably greater levels of CG than other samples. The 17Y and 21Y samples had considerably more EGCG than other samples. However, the concentration of EGC in the extract of teas with a short aging duration (19Y: 2-year-aged, 21Y: no aging) was substantially higher than in the other groups. 15Y had a considerably greater proportion of GC than other samples. Compared to 19Y or 21Y, GA content was greater in the lengthy aging period (15Y: 6-year-aged, 17Y: 4-year-aged). CAFF levels in sample 21Y were substantially greater than in other samples.

### The influence of aged black tea on fecal characteristics, gastrointestinal transit rate, and water intake

**Fig. 1:**
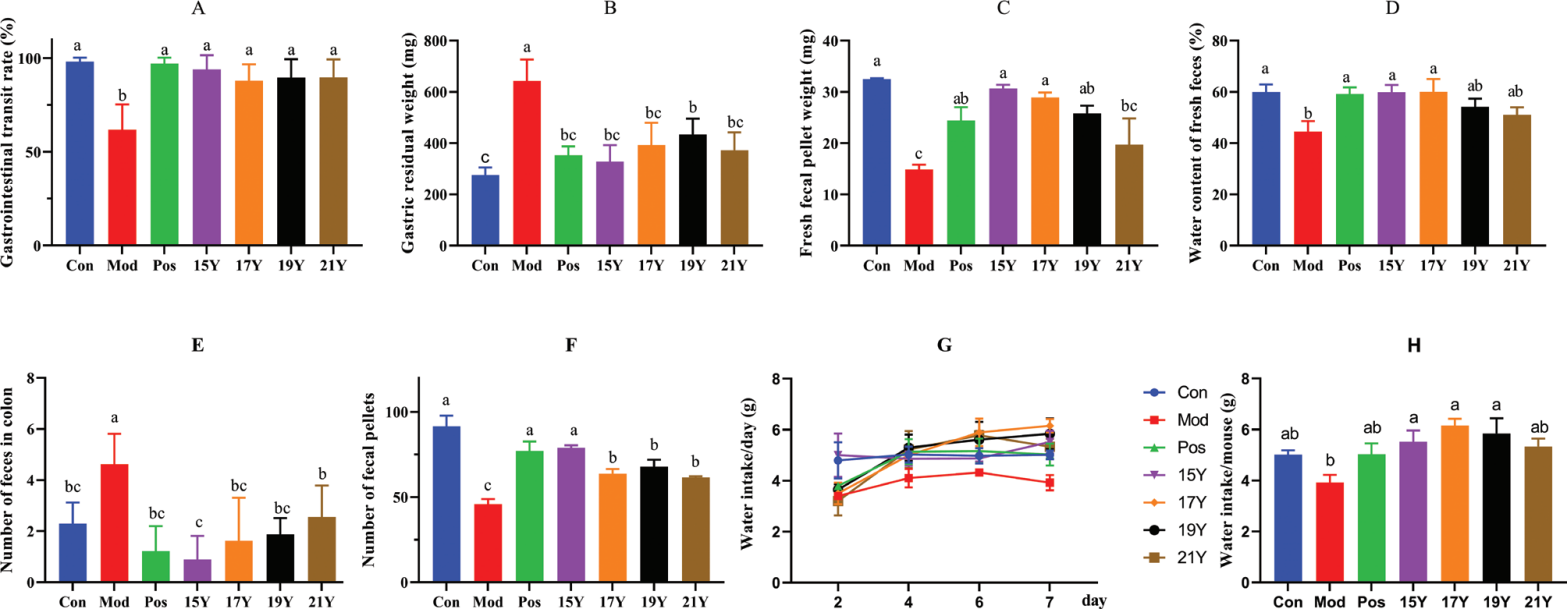
Gastrointestinal transit rate, defecation status and water intake of mice during the experiment. (A) Gastrointestinal transit rates after constipation treatment; (B) Residual weight in mouse stomach following India ink administration; (C) Fresh fecal weight; (D) Water content of fresh feces; (E) Numbers of formed feces remaining in the colon; (F) Converted into the amount of defecation per rat within 24 h; (G) Total amount of water intake per day during treatment; (H) Water intake per mouse on the last day of treatment. Con: normal control group; Mod: model group; Pos: Positive drug group; 15Y: aged 6 years black tea extract treatment group; 17Y: aged 4 years black tea extract treatment group; 19Y: aged 2 years black tea extract treatment group; 21Y: unaged black tea extract treatment group; Different letters (a, b, c) in the same row indicate significant differences between mean values (*P* < 0.05).

Figure 1 displays the findings of mice’s gastrointestinal transit rate, gastric residual weight, fresh fecal weight, fresh fecal water content, number of feces in the colon, and number of feces at 24 h. The gastrointestinal transit rate of mice was obtained by measuring the ratio of the distance the ink moved in the intestine to the length of the entire intestine of mice after gavage of India ink, as shown in Fig. 1A. When compared to the Con group, the Mod group of mice had considerably lower gastrointestinal transit rates; the four groups of black tea intervention mice’s gastrointestinal transit rates recovered and were substantially different from the Mod group and basically returned to normal compared with the Con group. The recovery of gastrointestinal transit rate was the best in 15Y group. The gastric residual weight of mice was obtained by measuring the weight of gastric contents after gavage of India ink, as shown in Fig. 1B. Mice in the Mod group gained considerably more gastric residual weight than mice in the Con group. Compared with the Mod group, the gastric residual mass of mice in the four groups of black tea intervention decreased significantly, among which 15Y, 17Y, and 21Y group all returned to normal level, and 15Y group had the best recovery. The fresh feces weight and fresh feces water content of mice were obtained by collecting fresh feces in clean cages and measuring their water content (Fig. 1C, D). The fresh feces weight and fresh feces water content of mice in the Mod group were significantly reduced compared with the Con group. Compared with the Mod group, the fresh feces weight and fresh feces water content of mice in the two groups (15Y and 17Y) with black tea intervention were significantly increased and both returned to normal levels. By extracting the colon from mice and calculating the amount of feces it contained, the quantity of feces in the colon was determined (Fig. 1E). The number of feces in the colon of mice in the Mod group was significantly increased compared with the Con group. The four groups with black tea intervention considerably decreased the amount of mouse feces in the colon compared to the Mod group, with 15Y group having the least amount. The mice were kept in clean cages so that the amount of feces they produced each day could be counted (Fig. 1F). The number of fecal pellets within 24 h from mice in the Mod group was significantly reduced compared with the Con group. Compared with the Mod group, the number of 24 h feces from mice in the four groups with black tea intervention was significantly increased, among which 15Y group was restored to the normal level. The drinking-water intake per day of the Mod group was reduced when compared with the Con group, but there was no significant difference, while those of the four groups with black tea intervention were increased diversely (Fig. 1G). As illustrated in Fig. 1H, there was no significant difference in water intake per mouse on the last day of treatment between the Mod group and the Con group, but 15Y, 17Y, and 19Y groups were significantly increased when compared with the Mod group. From the comprehensive view of the recovery effect, 15Y group showed the best effect, followed by 17Y group.

### Structure of colonic tissue and its effects on the use of aged black tea

**Fig. 2:**
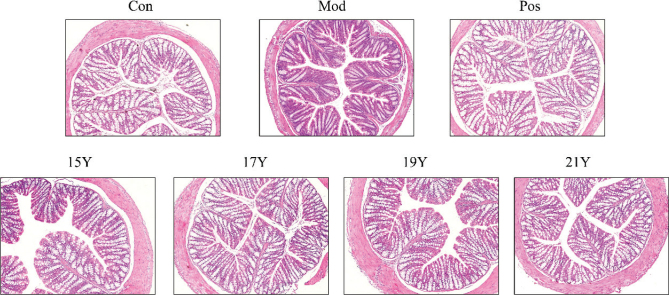
Histopathological observation of hematoxylin and eosin (H&E)-stained colon tissue slices (× 200).

Figure 2 displays a microscopic analysis of colonic tissue. In comparison to the Con group, the Mod group’s colon included a minimal amount of inflammatory cell infiltration, fewer goblet cells, shallower crypts, and a thinner colonic muscularis. After treatment of 15Y, 17Y, 19Y, or 21Y black tea extracts, the muscular and mucosal layers were clearly evident, and both crypts and goblet cells were plainly visible. However, no significant pathological alterations were identified in the black tea groups. Therefore, black tea extract can reduce the damage of loperamide hydrochloride to mouse colon tissue and maintain intestinal health.

### Blood serum level parameters after treatment with black tea extracts

**Fig. 3:**
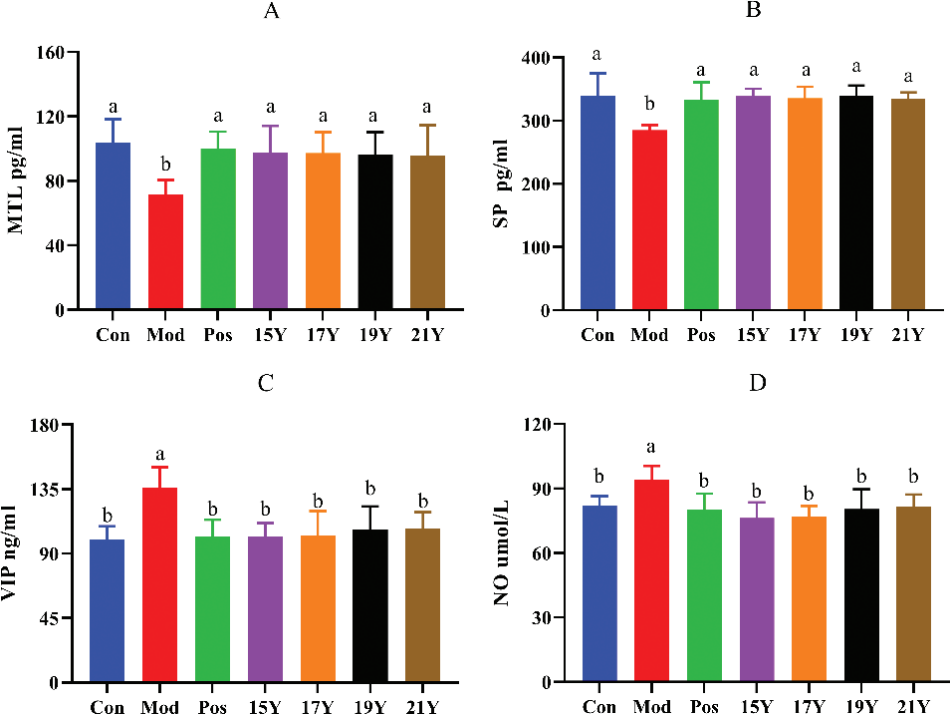
Effect of black tea on serum neurotransmitter levels in constipated mice. (A) MTL; (B) SP; (C) VIP; (D) NO; Different letters (a, b) in the same row indicate significant differences between mean values (*P* < 0.05).

In mouse serum, the intestinal excitatory neurotransmitters motilin (MTL) and substance P (SP) as well as the intestine inhibitory neurotransmitters VIP and NO activity were evaluated (Fig. 3). In contrast to the Con group, the Mod group’s serum MTL and SP activity levels were significantly lower than those of the Con group, but VIP and NO activity levels were substantially higher. After black tea intervention, the levels of MTL and SP activity were significantly elevated compared to the Mod group, whereas the levels of VIP and NO activity in the serum were dramatically reduced. The MTL, SP, VIP, and NO levels in the serum of the four black tea intervention groups were all returned to normal levels when compared to the Con group.

### Alterations in aquaporin gene and protein expression in the colon as a result of drinking aged black tea

**Fig. 4:**
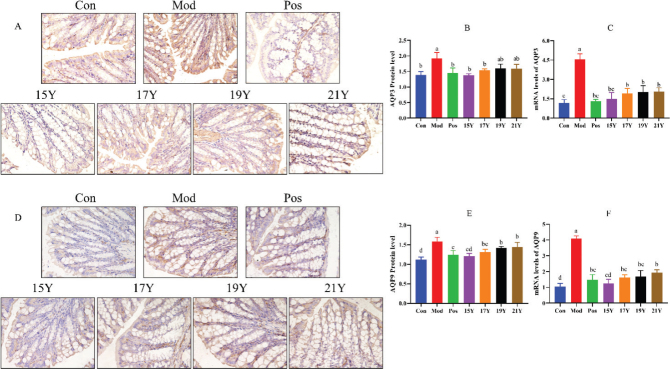
Effect of black tea on AQP3 and AQP9 expression in colon of constipated mice. (A) AQP3 expression level in colon tissue based on immunohistochemical analysis (original magnification, × 400); (B) AQP3 protein expression in colon tissue; (C) AQP3 gene expression in colon tissue; (D) AQP9 protein expression level in colon tissue based on immunohistochemical analysis (original magnification, × 400); (E) AQP9 protein expression in colon tissue; (F) AQP9 gene expression in colon tissue. Different letters (a, b, c, d) in the same row indicate significant differences between mean values (*P* < 0.05).

As illustrated in Fig. 4, an increase in AQP expression results in an increase in intestinal water absorption and/or a reduction in intestinal output. The findings for aquaporin protein expression matched those for aquaporin gene expression. The Mod group had considerably higher levels of aquaporin gene and protein expression than the Con group. The treatment of all the four black tea extracts dramatically decreased aquaporins gene and protein expression levels when compared to the Mod group, except AQP3 protein expression levels were not statistically different between 19Y, 21Y and Mod groups, but AQP3 gene expression levels were indeed statistically significant different of these two black tea administration groups from the Mod group. In particular, after the longest storage black tea (15Y) intervention, gene and protein expression of AQP3 and AQP9 were all returned to normal levels. Basically, with the increase of storage years, the expression of AQP3 and AQP9 in the colon of the black tea group showed a decreasing trend in different years.

### Correlation analysis of black tea components content and constipation indexes in constipated mice

**Fig. 5:**
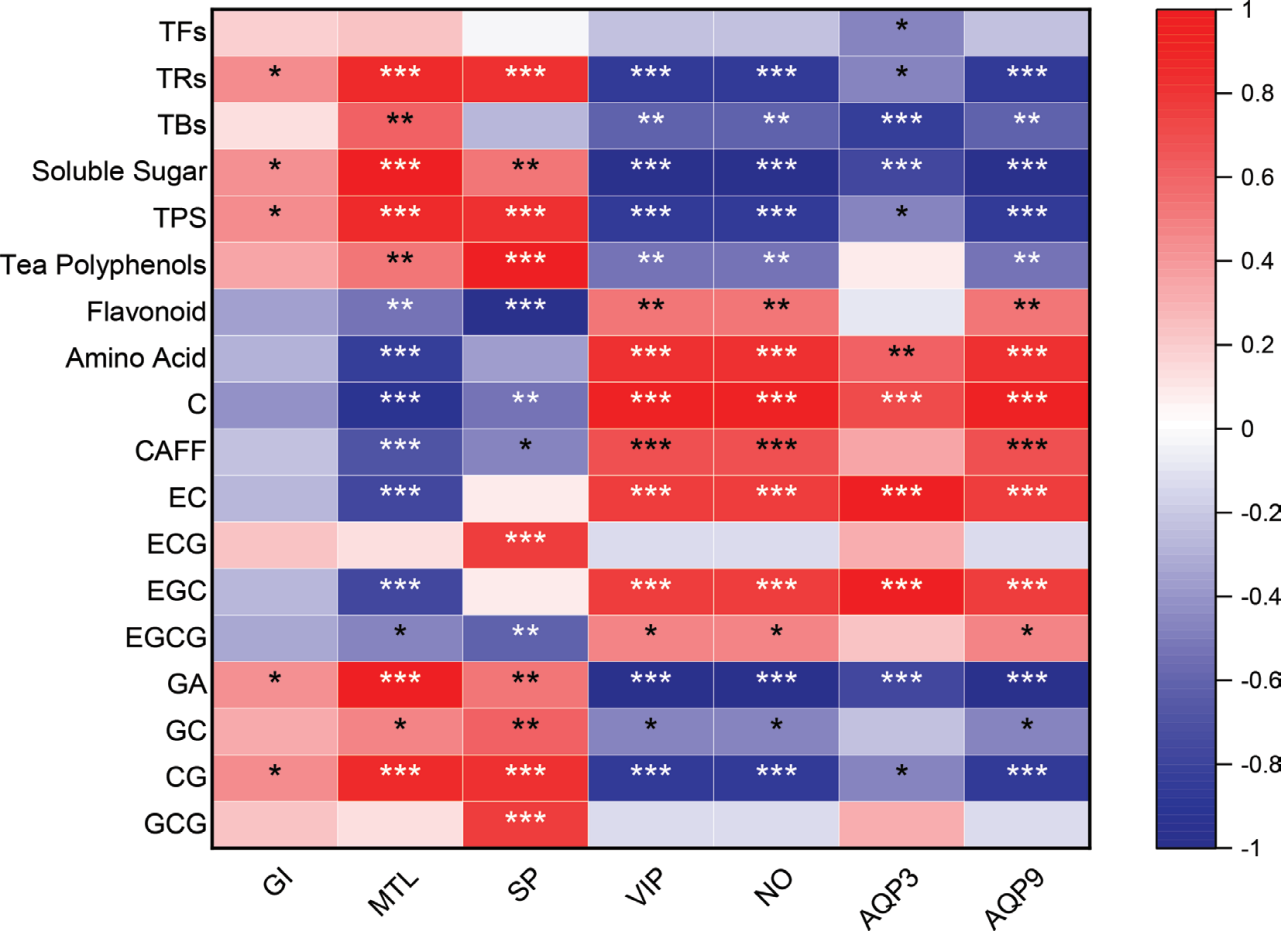
Correlation analysis of black tea components content and constipation indexes in constipated mice. Black tea samples came from four different tea samples including 15Y, 17Y, 19Y, and 21Y. Constipation indexes included gastrointestinal transit rate (GI), serum neurotransmitter levels (MTL, SP, VIP, NO) and AQP3, AQP9 expression levels in colon tissue (AQP3, AQP9). Black tea active ingredients include: Theaflavins (TFs), Thearubigins (TRs), Theabrownins (TBs), Total soluble sugar, Tea polysaccharides (TPS), Tea polyphenols, Flavonoid, Amino acids, Catechin (C), Caffeine (CAFF), Epicatechin (EC), Epig-allocatechin gallate (ECG), Epigallocatechin (EGC), Epigallocatechin gallate (EGCG), Gallic acid (GA), Gallocatechin (GC), catechin gallate (CG) and Gallocatechingallate (GCG). Spearman correlation analysis was performed. Red and blue squares indicated positively and negatively correlation, respectively. The *P*-value was calculated from the one-tailed option with a confidence interval of 95%. The statistically significant correlation was showed as *P < 0.05, **P < 0.01 and ***P < 0.001.

As shown in Fig. 5, the contents of TRs, soluble sugar, TPS, GA, and CG in black tea were significantly positively correlated with GI, and MTL and SP activity levels in serum, but significantly negatively correlated with VIP and NO activity levels in serum, as well as AQP3 and AQP9 expression levels in colon. Positive correlations were found between tea polyphenol and GC contents and MTL and SP activity levels in serum, while negative correlations were found between tea polyphenol and GC contents and VIP and NO activity levels in serum, as well as AQP9 expression levels in the colon. Black tea’s TBs content had a positive correlation with MTL activity level, a negative correlation with VIP and NO activity level in serum, as well as AQP3 and AQP9 expression levels in colon. Therefore, these compounds could be the active ingredients in black tea that effectively treat constipation.

## Discussion

Recent years have seen a rise in interest in the digestive system and a corresponding emphasis on intestinal health, with constipation being one of the most frequent gastrointestinal disorders (23). Compared with other gastrointestinal diseases, there are fewer types of drugs for constipation, many of which have negative side effects (24). In order to maintain proper bowel health, research has focused on adopting a safe, healthy diet without side effects.

Loperamide hydrochloride is mainly responsible for constipation by inhibiting intestinal peristalsis and then affecting intestinal transit as well as inhibiting the secretion of intestinal epithelial cells and increasing the reabsorption of water by the mucus layer (25). Constipation usually occurs when the weight of defecation is reduced, as a result of the difficulty in passing feces through the intestines, as well as the absorption of large amounts of water in the feces, the feces become hard and dry. We used a loperamide hydrochloride-induced constipation model in mice to simulate clinical constipation, and 10 days after loperamide hydrochloride (20 mg/kg, twice a day) modeling, fresh fecal weight and the amount of feces and fecal water content decreased in the Mod group, indicating that the modeling was successful. In the current study, we found that all of the black tea extract treatments had increased feces weight and fecal water content compared to the Mod group, with longer storage tea groups (15Y and 17Y) returning to normal levels and the mice showing a substantial improvement in their symptoms. An evaluation of the gastrointestinal transit rate is one way to detect gastrointestinal motility, which promotes the movement of intestinal contents. It is easier to excrete feces when the transit rate of the gastrointestinal tract is high. Otherwise, intestinal contents remain in the intestine for longer periods, resulting in impaired fecal excretion (26). The prolonged retention of feces in the intestinal tract can lead to a much lower-than-normal bowel movement frequency over a certain period. Our study showed that all four black tea extracts could significantly increase gastrointestinal transit rate. Previously reported studies have shown that oral administration of Pu-erh tea extract can improve the defecation weight, particle counts, and water content of defecation in constipation mice and increase the gastrointestinal transit rate (21), yellow tea extract can alleviate constipation symptoms by improving the fecal water content, defecation weight, and gastrointestinal transit rate, and extracts from dark tea can also significantly promoted gastrointestinal transit (8, 27). Our study revealed that black tea extract also can significantly alleviate constipation symptoms in mice.

The importance of colon health for defecation disorders and constipation is undeniable, the colon is able to accommodate varying volumes of intraluminal contents, it performs the last stage of digestion in the gastrointestinal system, including autonomic motor function, transport and fluid absorption, and storage of waste until defecation (28). Black tea extract was shown to protect colonic tissue by histologically examining the serosa, muscularis, submucosa, mucosa, crypts, and goblet cells.

Excitatory neurotransmitters like MTL and SP, as well as inhibitory ones like VIP and NO, are secreted by the enteric nervous system. It is believed that these neurotransmitters regulate intestinal function. In the present study, all four tea samples could increase excitatory neurotransmitter levels and reduce inhibitory neurotransmitter levels in constipated mice, and all of them could return to normal levels, thus resulting in the therapeutic effect of constipation, but there was no significant difference in the effect between the four tea samples. Li et al. found elevated levels of serum MTL, SP, gastrin (Gas), endothelin (ET), and acetylcholinesterase (AchE) in mice treated with different concentrations of Pu-erh tea compared with the constipation model group (21). Cao et al. found that yellow tea extract can lower VIP serum levels in constipation mice (8). Our study suggests that black tea extract also can modulate the release of intestinal neurotransmitters to relieve constipation.

The main function of the colon is to store feces, absorb water and electrolytes from the contents of the colon, and assist in defecation. AQPs are a group of transmembrane transporters with high selectivity for water and are expressed in multiple systemic tissues in the human body, so they are important for the regulation of water homeostasis (29). Previously, qRT-PCR indicated that yellow tea extract can reduce AQP3 and AQP4 mRNA expression (8). Our experimental results showed that the levels of AQP3 and AQP9 were significantly increased in the Mod group compared with the Con group. Thus, the amount of water in feces decreased in the Mod group although there was no significant difference in water intake. That implies that increasing the amount of water intake in mice was not enough to improve the water content of feces in constipated mice with high levels of aquaporins. In comparison with the Mod group, the expression level of AQPs in the colon was significantly lower after treatment with black tea extract. The results showed that with the increase of storage years, the expression of AQP3 and AQP9 in the colon of the black tea groups showed a decreasing trend in different years, 15Y group showed the best effect, followed by 17Y group. Therefore, the laxative mechanism of aged black tea may be through regulating (decreasing) the expression of AQP3 and AQP9 in the intestinal mucosa, so that the intestinal water reabsorption is reduced, the intestinal water is increased, the intestinal peristalsis is smooth, and the laxative effect is played.

The biochemical components of six categories of tea (green tea, white tea, yellow tea, oolong tea, black tea and dark tea) made of Yinghong NO.9 were different; black tea has more TRs and TBs than other teas (30). Published studies have reported that black tea extract can promote gastrointestinal motility; the mechanism of action includes cholinergic involvement and roles of prostaglandin and NO; and furthermore, the effective constituents TRs are found to be responsible for this effect (31). In addition, tea polyphenols are the most abundant active components in every type of tea, but as many as 90% of plant polyphenols escape digestion and absorption in the upper gastrointestinal tract and persist into the colon, where they come into contact with the gut microbiota and act as substrates for microbial production of small phenolic acids and short chain fat acids or actually affect gut microbial species composition and their metabolic activity (32). From the results of each index in the present study, all four black tea samples had the effect of improving constipation, this may be due to all four black tea samples containing effective ingredients to improve constipation. However, the specific active ingredients of black tea that exert anti-constipation effects remain unknown. 15Y had a considerably greater proportion of GC than other samples. Compared to 19Y or 21Y, GA content was greater in the lengthy aging period (15Y: 6-year-aged, 17Y: 4-year-aged). The results were consistent with the study of Huang et al. (33). Interestingly, 15Y group had the best effect on relieving constipation, followed by 17Y group. The longer the storage period, the better the effectiveness of black tea in relieving constipation. Meanwhile, with the increase in storage time, the composition of black tea changed, and the contents of TRs, soluble sugar, TPS, CG, GC, and GA in 15Y black tea were higher than those in the other three black tea samples. Correlation analysis of black tea components content and constipation indexes in constipated mice showed that TRs, soluble sugar, TPS, GA, and CG were all significantly positively associated with GI, excitatory neurotransmitters MTL and SP in serum, and all significantly negatively linked with inhibitory neurotransmitters VIP and NO in serum and water channel proteins AQP3 and AQP9 expression in colon. Thus, it was speculated that TRs, soluble sugar, TPS, GA, and CG may be the effective constituents in aged black tea responsible for alleviating constipation.

## Conclusions

In this study, we found that mice constipation produced by loperamide hydrochloride may be alleviated by aged black tea. On the one hand, constipated mice treated with black tea extract may experience an increase in the number of defecations, fecal water content, gastrointestinal transit rate, and blood concentrations of excitatory neurotransmitters MTL and SP. On the other hand, treatment with black tea extract could promote gastric emptying to reduce colonic fecal accumulation, decreased levels of inhibitory neurotransmitters VIP and NO, and downregulated expression levels of aquaporins (AQP3 and AQP9). Aged black tea with the longest storage (15Y) had the best effect on relieving constipation. Soluble sugar, TPS, tea polyphenols, TRs, CG, and GA were increased in the other three black tea samples compared to the new tea (21Y). Correlation analysis showed that the therapeutic efficacy of black tea samples on constipation might be related to contents of TRs, soluble sugar, TPS, GA, and CG. These results could indicate that aged black tea could be used as an effective dietary strategy to relieve constipation. But this experiment also gives some thought that the oldest tea samples used in this experiment are only 6 years old, so will tea stored for longer years work better? In addition, since gut microbiota plays an important role in intestinal function, whether the activity of aged black tea to ease constipation is related to regulate gut microflora also needs to be further studied.

## Authors’ contributions

Y. Wu wrote the manuscript; Y. Wu and Q. Li performed the research and analyzed the data; J. Cao, D. Li, and S. Sun designed and funded the experiments; F. Fan, R. Wu, and J. Jin contributed to animal study and data analysis; R. Chen and L. Sun participated in H&E and qPCR; Z. Zhang and X. Lai participated in HPLC and IHC; L. Gan, W. Wong and D. Li revised the manuscript. All authors read and approved the final manuscript.

## Conflict of interest and funding

The authors declare that they have no competing interests. This study was funded by the “Agricultural competitive industry discipline team building project of Guangdong Academy of Agricultural Sciences” [Grant No.: 202126TD]; Qingyuan Science and Technology Plan Projects [Grant No.: 2021ZDZX002]; Food nutrition and health Collaborative Innovation Center of GDAAS [Grant No.: XT202229]; Guangdong Basic and Applied Basic Research Foundation [Grant Nos.: 2020A1515011266, 2021A1515010958]; Guangzhou Science and Technology Plan Projects [Grant Nos.: 202102020047, 202002030202, 202201011455]; Key-Area Research and Development Program of Guangdong Province [Grant No.: 2020B0202080003]; Innovation Fund projects of Guangdong Academy of Agricultural Sciences (Grant Nos.: 202115, 202035); Maoming Science and Technology Program (Grant No.: mmkj2020045); Zhanjiang Science and Technology Program (Grant No.: 2020A03014); Innovation Fund projects of Guangdong Academy of Agricultural Sciences (Grant No.: 202115, 202035); Special fund for scientific innovation strategy-construction of high level Academy of Agriculture Science (Grant No.: R2019PY-JX004); the Innovation Fund projects of Guangdong Key Laboratory of Tea Plant Resources Innovation and Utilization (Grant No.: 2021CX02); Special fund project for introduction of scientific and technological talents of Guangdong Academy of Agricultural Sciences (Project No.: R2021YJ-YB3014).

## Supplementary Material


