# Supplementary material for: Aged black tea alleviates constipation in mice by modulating intestinal neurotransmitters and decreasing AQP3 and AQP9 expression
Source: Food Nutr Res. 2023 Oct 30;67:10.29219/fnr.v67.9513. doi: 10.29219/fnr.v67.9513 (PMC11801384; doi:10.29219/fnr.v67.9513)
Supplement: Supplementary file 1 [file FNR-67-9513-s001.zip › 9513-Supplementary Material-68799-1-11-20230730.docx]

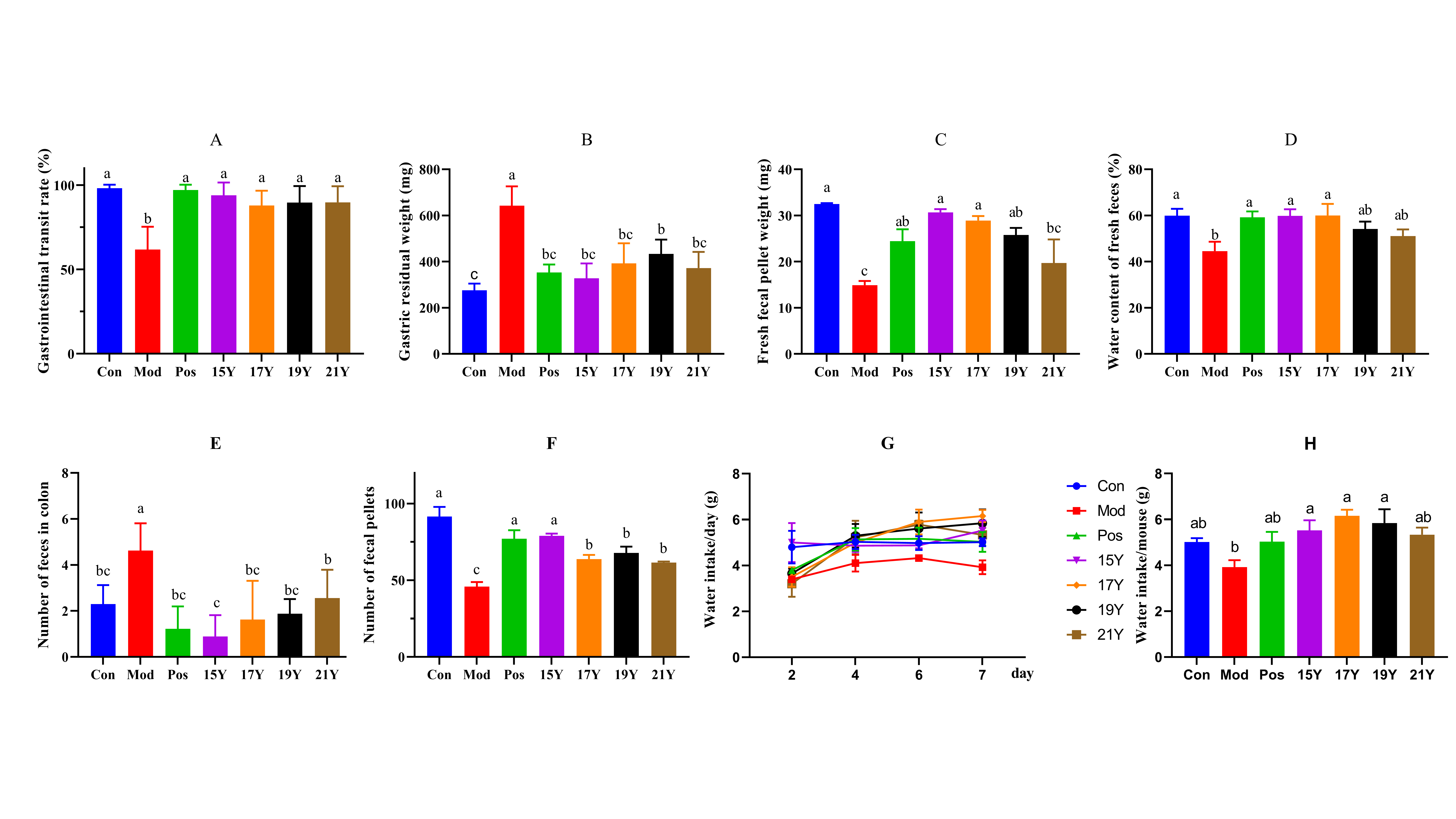


**Figure 1 Gastrointestinal transit rate, defecation status and water intake of mice during the experiment.**

(A) Gastrointestinal transit rates after constipation treatment; (B) Residual weight in mouse stomach following India ink administration; (C) Fresh fecal weight; (D) Water content of fresh feces; (E) Numbers of formed feces remaining in the colon; (F) Converted into the amount of defecation per rat within 24 hours; (G) Total amount of water intake per day during treatment; (H) Water intake per mouse on the last day of treatment. Con: normal control group; Mod: model group; Pos: Positive drug group; 15Y: aged 6 years black tea extract treatment group; 17Y: aged 4 years black tea extract treatment group; 19Y: aged 2 years black tea extract treatment group; 21Y: unaged black tea extract treatment group; Different letters (a, b, c) in the same row indicate significant differences between mean values (*p* < 0.05)


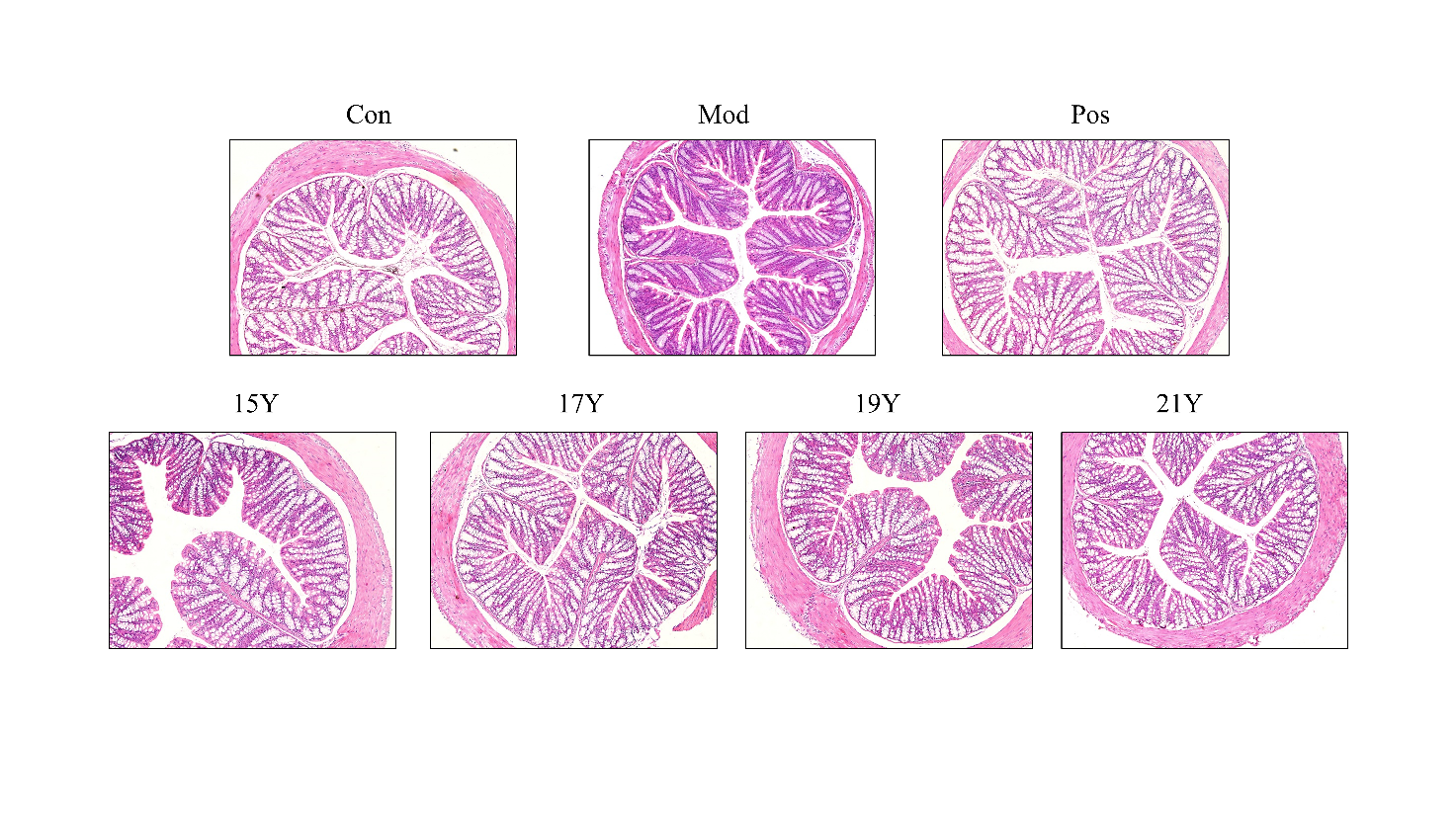
**Figure 2 Histopathological observation of hematoxylin and eosin (H&E)-stained colon tissue slices (× 200)**


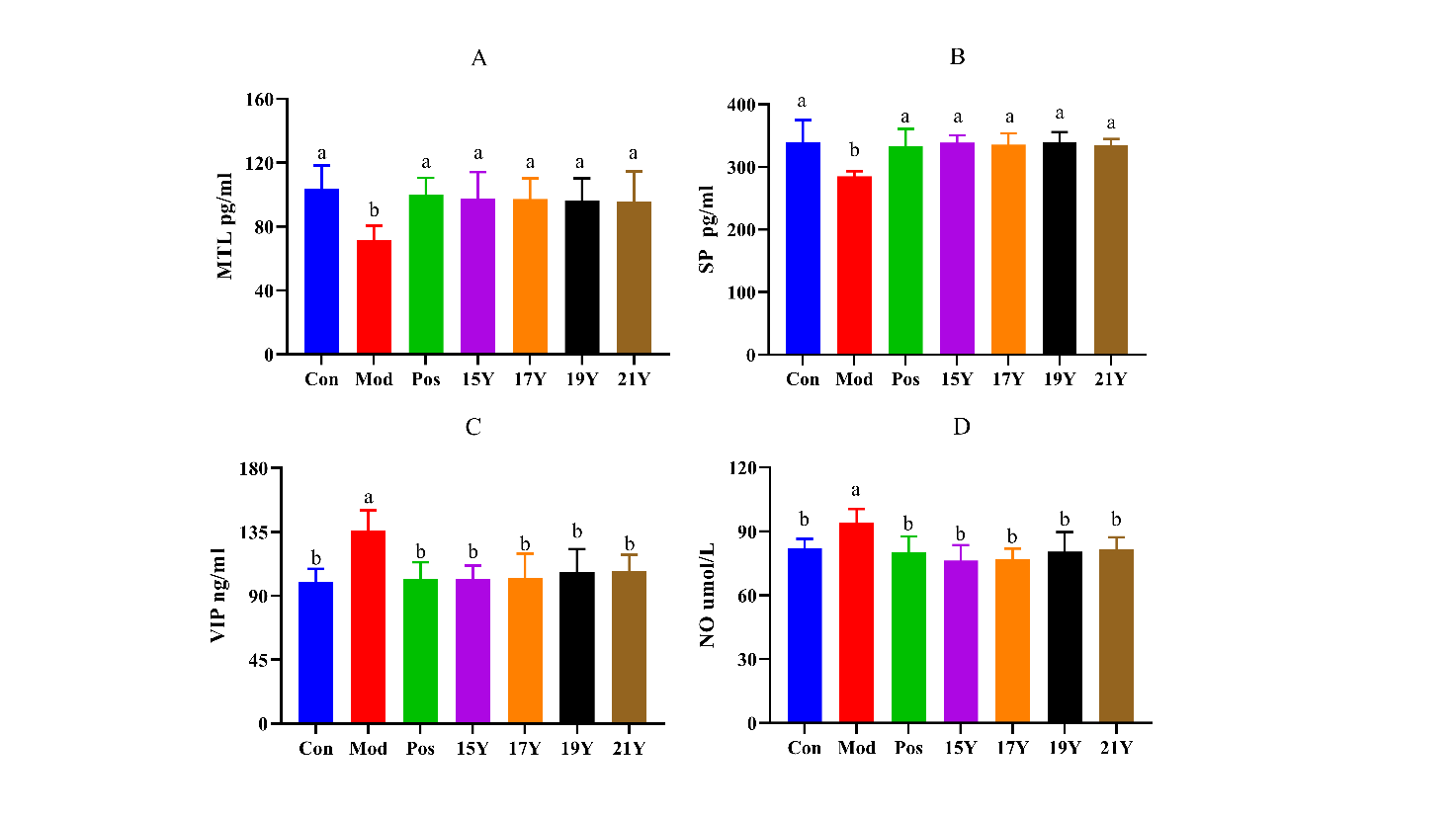


**Figure 3 Effect of black tea on serum neurotransmitter levels in constipated mice.**

(A) MTL; (B) SP; (C) VIP; (D) NO; Different letters (a, b) in the same row indicate significant differences between mean values (*p* < 0.05)


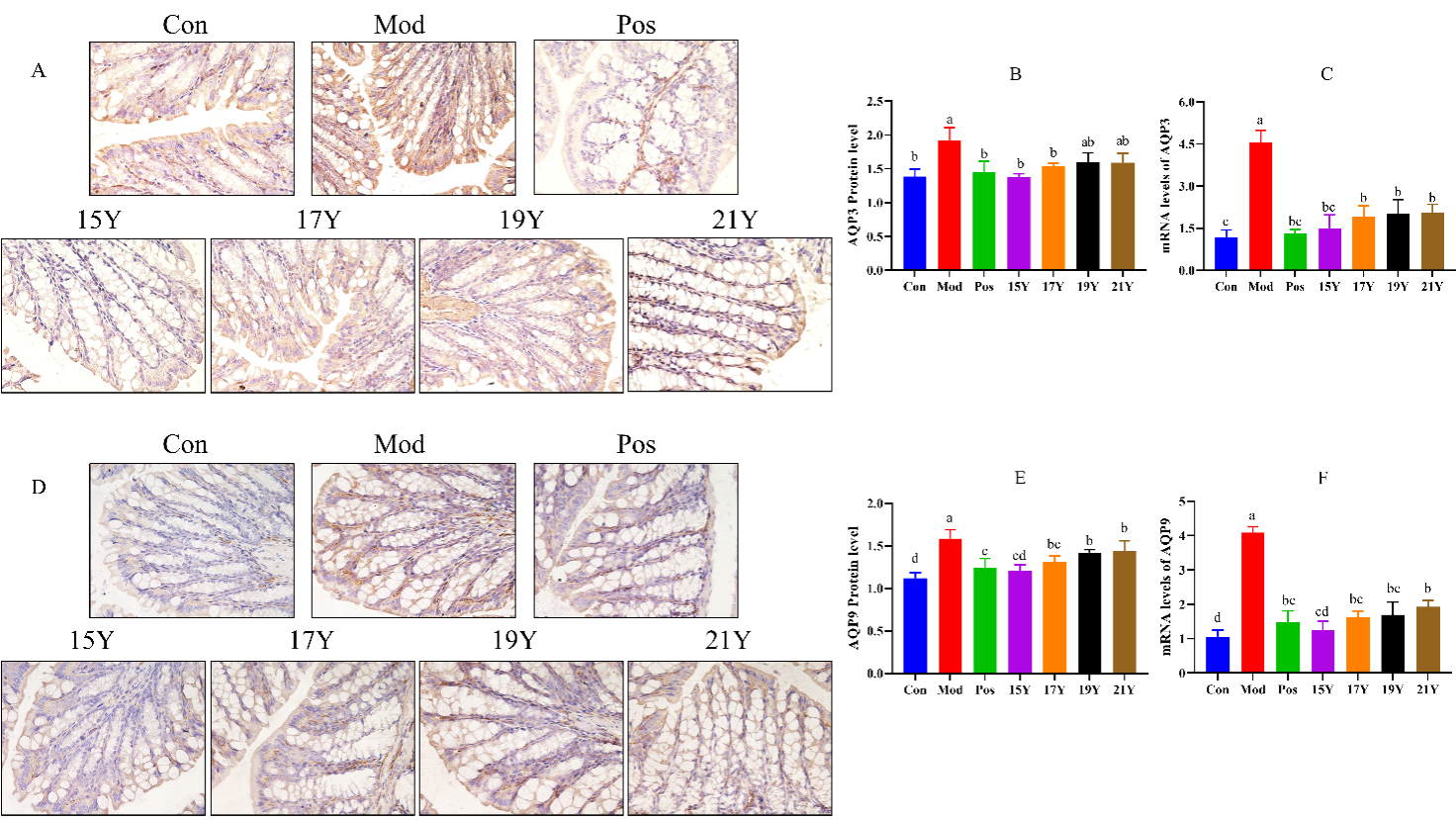


**Figure 4 Effect of black tea on AQP3 and AQP9 expression in colon of constipated mice.**

(A) AQP3 expression level in colon tissue based on immunohistochemical analysis (original magnification, × 400); (B) AQP3 protein expression in colon tissue; (C) AQP3 gene expression in colon tissue; (D) AQP9 protein expression level in colon tissue based on immunohistochemical analysis (original magnification, × 400); (E) AQP9 protein expression in colon tissue; (F) AQP9 gene expression in colon tissue. Different letters (a, b, c, d) in the same row indicate significant differences between mean values (*p* < 0.05)

**Figure 5 Correlation analysis of black tea components content and constipation indexes in constipated mice.**

Black tea samples came from four different tea samples including 15Y, 17Y, 19Y, and 21Y. Constipation indexes included gastrointestinal transit rate (GI), serum neurotransmitter levels (MTL, SP, VIP, NO) and AQP3, AQP9 expression levels in colon tissue (AQP3, AQP9). Black tea active ingredients include: Theaflavins (TFs), Thearubigins (TRs), Theabrownins (TBs), Total soluble sugar, Tea polysaccharides (TPS), Tea polyphenols, Flavonoid, Amino acids, Catechin (C), Caffeine (CAFF), Epicatechin (EC), Epig-allocatechin gallate (ECG), Epigallocatechin (EGC), Epigallocatechin gallate (EGCG), Gallic acid (GA), Gallocatechin (GC), catechin gallate (CG) and Gallocatechingallate (GCG). Spearman correlation analysis was performed. Red and blue squares indicated positively and negatively correlation, respectively. The *p*-value was calculated from the one-tailed option with a confidence interval of 95%. The statistically significant correlation was showed as **p* < 0.05, ***p* < 0.01 and ****p* < 0.001
