# Supplementary material for: Aged black tea alleviates constipation in mice by modulating intestinal neurotransmitters and decreasing AQP3 and AQP9 expression
Source: Food Nutr Res. 2023 Oct 30;67:10.29219/fnr.v67.9513. doi: 10.29219/fnr.v67.9513 (PMC11801384; doi:10.29219/fnr.v67.9513)
Supplement: Supplementary file 1 [file FNR-67-9513-s001.zip › 9513-Supplementary Material-68800-1-11-20230730.docx]

**Table 1 Nucleotide sequences of primers**

| Gene name | Sequence (5′–3′) | Length/bp |
| --- | --- | --- |
| AQP3 | Forward: GCTGTGACCTTCGCAATGTG  Reverse: CAAAGATTCCAGCTGTGCCG | 20 |
| AQP9 | Forward: CGAGAAAAGGCTGGTGGGAT  Reverse: AAAAGACACCGCTGGGTTGA | 20 |
| GAPDH | Forward: GGAGAGTGTTTCCTCGTCCC  Reverse: ACTGTGCCGTTGAATTTGCC | 20 |

**Table 2 Contents (%) of main phytochemical components in black tea samples^a^**

| Component | 15Y | 17Y | 19Y | 21Y |
| --- | --- | --- | --- | --- |
| Water | 11.05 ± 0.05 ^a^ | 10.33 ± 0.05 ^b^ | 8.62 ± 0.06 ^d^ | 9.41 ± 0.02 ^c^ |
| Water extract | 37.78 ± 0.1 ^a^ | 36.3 ± 0.01 ^b^ | 36.48 ± 0.07 ^b^ | 34.97 ± 0.18 ^c^ |
| Flavonoids | 1.47 ± 0.01 ^c^ | 1.56 ± 0.03 ^b^ | 1.40 ± 0.02 ^d^ | 1.72 ± 0.00 ^a^ |
| Amino acids | 2.26 ± 0.02 ^c^ | 1.9 ± 0.02 ^d^ | 2.6 ± 0.07 ^b^ | 2.71 ± 0.05 ^a^ |
| Soluble sugar | 7.47 ± 0.09 ^a^ | 6.97 ± 0.06 ^b^ | 6.5 ± 0.00 ^c^ | 6.26 ± 0.02 ^d^ |
| Tea polysaccharides | 1.01 ± 0.09 ^a^ | 0.86 ± 0.04 ^b^ | 0.93 ± 0.04 ^ab^ | 0.86 ± 0.02 ^b^ |
| Tea polyphenols | 12.77 ± 0.03 ^b^ | 12.39 ± 0.07 ^c^ | 16.87 ± 0.00 ^a^ | 11.36 ± 0.1 ^d^ |
| Theaflavins | 0.13 ± 0.00 ^a^ | 0.10 ± 0.00 ^b^ | 0.11 ± 0.01 ^ab^ | 0.11 ± 0.01 ^ab^ |
| Thearubigins | 2.19 ± 0.01 ^a^ | 1.63 ± 0.17 ^c^ | 1.70 ± 0.02 ^b^ | 1.42 ± 0.01 ^d^ |
| Theabrownins | 3.35 ± 0.06 ^a^ | 3.46 ± 0.03 ^a^ | 2.69 ± 0.03 ^c^ | 2.93 ± 0.03 ^b^ |

**^a^**Values represent mean ± SD (n = 3). Different letters (a, b, c, d) in the same row indicate significant differences between mean values (*p* < 0.05).

**Table 3 Contents (mg/g) of catechin and caffeine monomers in black tea samples^a^**

| Component | 15Y | 17Y | 19Y | 21Y |
| --- | --- | --- | --- | --- |
| C | 0.896 ± 0.134 ^c^ | 1.082 ± 0.012 ^bc^ | 2.3 ± 0.061 ^a^ | 1.25 ± 0.062 ^b^ |
| CG | 1.979 ± 0.336 ^a^ | 0.561 ± 0.035 ^b^ | 1.617 ± 0.017 ^a^ | 0.52 ± 0.002 ^b^ |
| EC | 1.475 ± 0.065 ^c^ | 1.791 ± 0.032 ^c^ | 5.827 ± 0.165 ^a^ | 2.183 ± 0.159 ^b^ |
| ECG | 6.69 ± 1.113 ^b^ | 5.655 ± 0.008 ^b^ | 15.433 ± 0.047 ^a^ | 5.66 ± 0.061 ^b^ |
| EGC | 3.26 ± 0.12 ^c^ | 5.8 ± 0.29 ^b^ | 8.11 ± 0.4 ^a^ | 7.95 ± 0.17 ^a^ |
| EGCG | 5.01 ± 0.143 ^c^ | 7.679 ± 0.003 ^a^ | 5.662 ± 0.37 ^b^ | 7.286 ± 0.058 ^a^ |
| GC | 56.964 ± 1.894 ^a^ | 15.516 ± 0.235 ^c^ | 52.181 ± 0.245 ^b^ | 16.629 ± 0.112 ^c^ |
| GCG | 2.007 ± 0.18 ^b^ | 1.725 ± 0.003 ^b^ | 2.383 ± 0.021 ^a^ | 1.85 ± 0.089 ^b^ |
| GA | 1.93 ± 0.094 ^a^ | 1.756 ± 0.047 ^a^ | 1.483 ± 0.049 ^b^ | 1.248 ± 0.072 ^c^ |
| CAFF | 55.92 ± 1.4 ^b^ | 54.47 ± 0.03 ^b^ | 55.92 ± 0.3 ^b^ | 59.047 ± 0.729 ^a^ |

**^a^**Dissolve gallic acid (GA), gallocatechin (GC), epigallocatechin (EGC), catechin (C), caffeine (CAFF), epicatechin (EC), epigallocatechin gallate (EGCG), gallocatechingallate (GCG), epigallocatechin gallate (ECG), catechin gallate (CG); Values represent mean ± SD (n = 3). Different letters (a, b, c, d) in the same row indicate significant differences between mean values (*p* < 0.05).
