# Supplementary figures and images for: Aged black tea alleviates constipation in mice by modulating intestinal neurotransmitters and decreasing AQP3 and AQP9 expression
Source: Food Nutr Res. 2023 Oct 30;67:10.29219/fnr.v67.9513. doi: 10.29219/fnr.v67.9513 (PMC11801384; doi:10.29219/fnr.v67.9513)

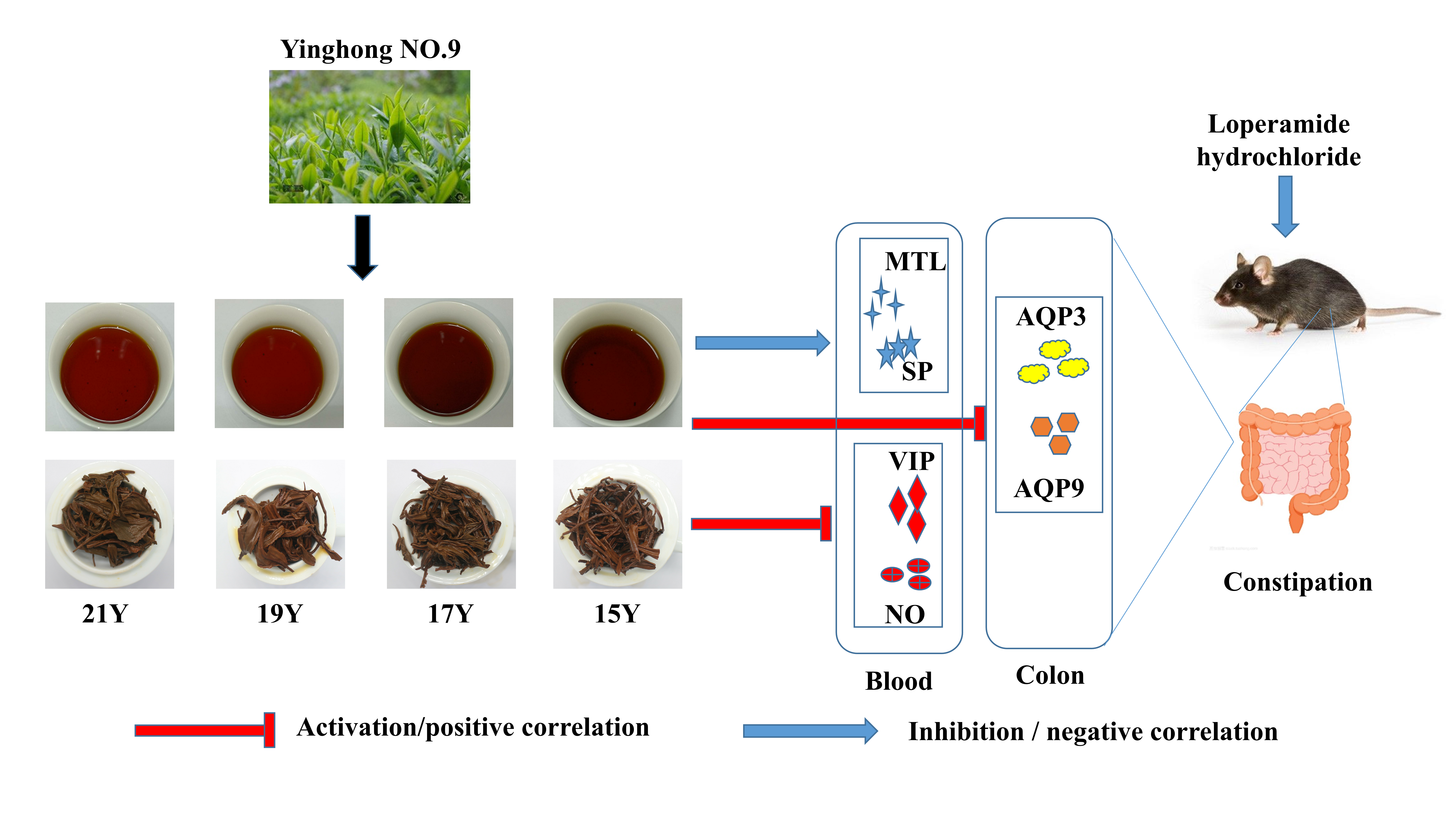

Supplement: Supplementary file 1 [file FNR-67-9513-s001.zip › 9513-Supplementary Material-68801-1-11-20230730.tif]
